# Average Daily Gain in Lambs Weaned at 60 Days of Age Is Correlated with Rumen and Rectum Microbiota

**DOI:** 10.3390/microorganisms11020348

**Published:** 2023-01-30

**Authors:** Xuejiao Yin, Chunhui Duan, Shoukun Ji, Peizhi Tian, Sisi Ju, Hui Yan, Yingjie Zhang, Yueqin Liu

**Affiliations:** College of Animal Science and Technology, Hebei Agricultural University, Baoding 071000, China

**Keywords:** rumen, rectum, microbiota, average daily gain, weaned lambs

## Abstract

Colonization of gastrointestinal microbiota in mammals during early life is vital to host health. The objective of this study was to investigate whether lambs with high and low ADG have a different rumen and rectum microbial community. Thus, we investigated potential relationships between rumen and rectum microbiota and average daily gain (ADG) in weaned lambs. Sixteen lambs with similar body weights (7.63 ± 1.18 kg) were selected at 30 days of age. At 60 days of age, lambs were weaned, and ADG was calculated from 60 to 90 days. Then, two groups were generated: higher ADG (HG, 134.17 ± 13.48 g/day) and lower ADG (LG, 47.50 ± 19.51 g/day). Microbiota was evaluated at 30, 60, and 90 days of age. The final live weight and ADG at 90 days of age was higher (*p* < 0.05) in the HG group compared to the LG group. The maturity of bacterial and fungal communities was increased (*p* < 0.05) in the HG group for the 30 days vs. 90 days comparison and 60 days vs. 90 days comparison. Linear discriminant analysis effect size (LEfSe) analysis revealed a total of 18 bacterial biomarkers that are ADG-specific in the rumen and 35 bacterial biomarkers in the rectum. Meanwhile, 15 fungal biomarkers were found in the rumen and 8 biomarkers were found in the rectum. Our findings indicated that ADG is related to the rumen and rectum microbiota in lambs.

## 1. Introduction

Gastrointestinal microbiota has been demonstrated to be critical for host health by providing metabolic products, maintaining metabolic function, developing the immune system, and defending against pathogens [1,2,3]. Furthermore, the gut microbiota serves as a habitat for commensal bacteria, which act as an additional defense barrier [4]. Particularly, the early life establishment of lambs’ gut microbiota sets the stage for the adult microbiome and has prolonged influence on host health [5,6]. Therefore, an ecological balance of microorganisms in the gut increases the host’s resistance to disease and assures optimal function of the gastrointestinal tract [7].

Weaning is stressful for animals due to adapting to solid feed, coping with maternal separation, and establishing a social hierarchy [8]. During this period, the development of the digestive system in lambs is incomplete [9] and much of the imbalance of homeostasis associated with weaning stress is related to it [10]. Weaning stress could disrupt intestinal barrier integrity and disturb the ecological balance of the gut microbial community [11]. The physiological and psychological stress of weaning will influence the metabolism [12], ruminal microflora [13], and immune response [14].

Although the effects of weaning age [7,15], additive [16], and weaning method [14] have been extensively studied, few studies have focused on the development of gut microbiota during the weaning period [17,18,19]. The average daily gain (ADG) is an important indicator for identifying weaning stress in animals [20,21]. Recently, more and more studies not only focus on the modification of gastrointestinal microbiota [14,22] but also on the relationship between the host performance and the microbial composition [23,24]. The gastrointestinal microbiota was related to the feed efficiency in cattle [25], diarrhea in calves [26], the milk production of dairy cows [23], and the stillbirth rate of sows [27]. However, there are no studies focused on the difference in gastrointestinal microbiota between lambs with low and high ADG during the weaning period.

The aim of this study was to discover the specific microbial taxa in the gastrointestinal tract that could be connected with ADG in weaned lambs. Here, we compared the differences of microbial communities in lambs raised in the same condition; the lambs have similar body weight and ADG before weaning, but after weaning, the lambs show different body weight and ADG. Our hypothesis was that there was a different rumen and rectum microbial community in high- and low-ADG weaned lambs. Our findings will provide a better understanding of the influence of rumen and rectum microbiota on the ADG of lambs during the weaning period, and a framework for future studies on increasing the growth performance of lambs.

## 2. Materials and Methods

### 2.1. Experimental Animals and Diets

The animal study was reviewed and approved by the Animal Care and Use Committee of Hebei Agricultural University (Project ID: YJ201825).

A total of 16 30-day-old female lambs (Hu sheep, singleton) were selected as experimental animals (body weight = 7.63 ± 1.18 kg, mean ± SE) from the sheep industry test station (Hengshui, China). These lambs were raised according to the conventional system used in our experimental facility. Before weaning, the lambs were housed with their mothers in mother–lamb combined pens and had free access to a commercial starter feed from day 15 (Supplementary Table S1). On day 60, the lambs were weaned off their dams and were moved to other pens. All experimental lambs were kept in individual pens (3.0 × 0.8 m). After weaning, all lambs had free access to fresh water and were fed two times daily (0730 and 1500 h) with a total mixed ration, as shown in Supplementary Table S1. No animals were treated with antibiotics throughout the study.

### 2.2. Grouping and Sampling Period

The experimental period was from 30 to 90 days of age. The lambs were weighed before the morning feeding at 30, 60, and 90 days of age, while ADG was calculated between 30 to 60 days of age and 60 to 90 days of age. The ADG was 194 ± 32.04 and 90.83 ± 64.04 (mean ± SD), between 30 to 60 days and 60 to 90 days, respectively. Thus, according to the high deviation in ADG after weaning, we found the lambs have different ADG in this period. Therefore, at the end of this period, we ranked their ADG during the 30 days after weaning (60 to 90 days of age) and divided the 16 lambs into two groups according to the ranking list: eight lambs with lower ADG (LG, 47.50 ± 19.51 g/day), and eight lambs with higher ADG (HG, 134.17 ± 13.48 g/day).

### 2.3. Samples Collection and Measurement

Ruminal digesta samples were collected from each lamb using a flexible polyvinyl chloride (PVC) tube 2 h after the morning feeding at 30, 60, and 90 days of age. The PVC tube was thoroughly cleaned with fresh warm water between sample collections, and the first 20 mL of collected rumen fluid was discarded to avoid contamination from saliva. Fecal samples were collected from the rectum by manual extraction using sterile gloves and sterile cotton swabs at 30, 60, and 90 days of age. All samples were placed in sterile containers, snap frozen in liquid nitrogen immediately, and then stored at −80 °C until DNA extraction.

### 2.4. DNA Extraction and Sequencing

Five lambs were randomly chosen in each experimental group. Total genomic DNA of the rumen bacteria was extracted from 2 mL of rumen liquid and 0.5 g of the rectal digesta samples using the OMEGA Stool DNA Kit (Omega Bio-tek, Inc., Norcross, GA, USA) according to the manufacturer’s specifications. The quality of the obtained DNA was confirmed by 1% agarose gel electrophoresis and spectrophotometer (optical density at 260/280 nm ratio). The amplicon library preparation was performed by polymerase chain reaction (PCR) amplification of the V3-V4 hypervariable regions of the bacterial 16S rRNA gene using the primers 338F (5′-CTCCTACGGGAGGCAGCAG-3′) and 806R (5′-GGACTACHVGGGTWTCTAAT-3′). The ITS rRNA genes were amplified with the primer ITS1F (5′-CTTGGTCATTTAGAGGAAGTAA-3′) and ITS2R (5′-GGACTACNNGGGTATCTAAT-3′). For each sample, 8-nucleotide barcode sequences that were unique to each sample were added to the 5′ end of the forward and reverse primers (provided by Allwegene Company, Beijing, China). PCRs were performed in 25 μL reaction volumes containing 12.5 μL KAPA 2G Robust Hot Start Ready Mix, 1 µL of each primer (5 µM), 5 µL DNA (total template quantity was 30 ng), and 5.5 µL H_2_O. The thermocycling protocol of the amplification was as follows: 5 min of denaturation at 95 °C, followed by 32 cycles of 95 °C for 45 s, 50 s for annealing at 55 °C, and 45 s for elongation at 72 °C with a final extension at 72 °C for 10 min. The amplified PCR products were visualized on 1% agarose gels, purified using a QIAquick Gel Extraction Kit (QIAGEN, Hilden, Germany), and quantified using QuantiFluor™-ST (Promega, Madison, WI, USA). After that, the purified PCR products were pooled in equimolar amounts and sequenced on an Illumina Miseq paired-end 300 sequencing platform at the Allwegene Company (Beijing, China).

### 2.5. Bioinformatics Analysis

Shorter reads (≤300 bp) with low quality scores (≤20) and reads containing ambiguous bases or those that did not exactly match primer sequences and barcode tags were filtered out from the dataset using QIIME [28]. Clean sequences with an overlap longer than 10 bp were assembled using FLASH-1.2.11 [29]. Reads that could not be assembled were discarded. Chimera sequences were detected using usearch6.1 [30]. Qualified reads were separated using sample-specific barcode sequences and trimmed with Illumina Analysis Pipeline Version 2.6. The resulting dataset was analyzed using QIIME. The remaining sequences were clustered into operational taxonomic units (OTUs) at a 97% similarity using the Ribosomal Database Project (RDP) Classifier tool [31].

The OTU level alpha diversity of bacterial communities was determined using Chao 1 and Shannon indices and calculated by QIIME. Principal Coordinates Analysis (PCoA) plots of the Bray-Curtis metric were calculated with square root transformed data using vegan package in R (version 4.2.0) software. Heat maps were generated using the package “pheatmap” of R software.

### 2.6. Statistical Analysis

All statistical analyses were carried out using R (version 4.2.0) software. The individual lambs served as an experimental unit. Before analysis, the normal distribution of data was confirmed by the Shapiro-Wilk’s test. Variation in body weight and average daily gain was examined by the *t*-test. The dissimilarities of microbiota between LG and HG groups were evaluated using the Mann-Whitney U test. Analysis of similarities (ANOSIM, vegan package) (999 permutations) using Bray-Curtis distances was performed to compare the similarity of microbial communities among the observed microbial profiles. To identify the specific microbial taxa associated with different ADG groups, we performed a comparison of the ruminal and rectal microbiota using the linear discriminant analysis (LDA) effect size (LEfSe) with default parameters (LDA score > 2 and *p* < 0.05), which would allow the discovery of biomarkers [32]. Correlation between parameters was performed by using Spearman’s correlation (psych package). *p*-value <0.05 was considered statistical significance.

## 3. Results

### 3.1. Growth Performance

**Figure 1:**
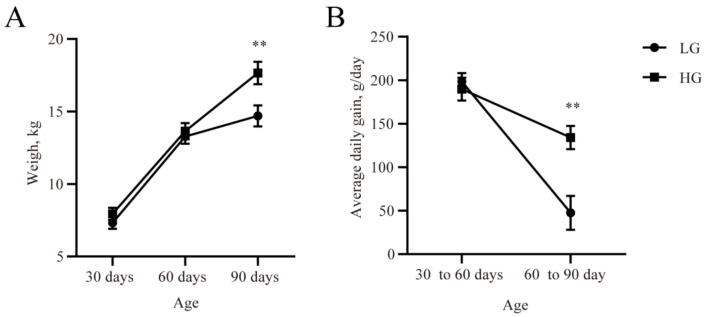
The live weight (**A**) and average daily gain at different lamb ages (**B**). Data are expressed as mean ± SE. ** *p* < 0.05. LG: the weaned lambs with lower average daily gain. HG: the weaned lambs with higher average daily gain.

The body weight and ADG of the two groups is given in Figure 1. Although the live weights at 30 and 60 days were similar across the two groups, the final live weight at 90 days of age was lower (*p* < 0.05, Figure 1A) in the LG group. ADG was lower (*p* < 0.05, Figure 1B) in the LG group in comparison with the HG group during the 30 days after weaning.

### 3.2. Differences in the Rumen and Rectum Microbiota Community of LG and HG Lambs before Weaning

**Figure 2:**
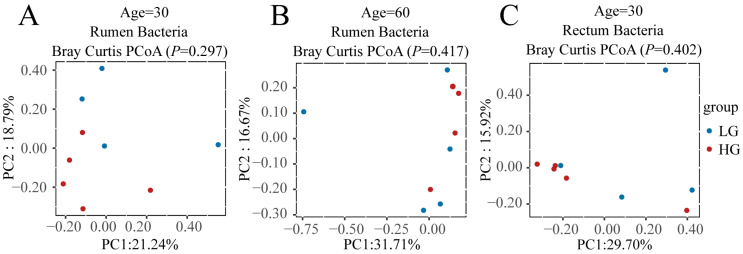

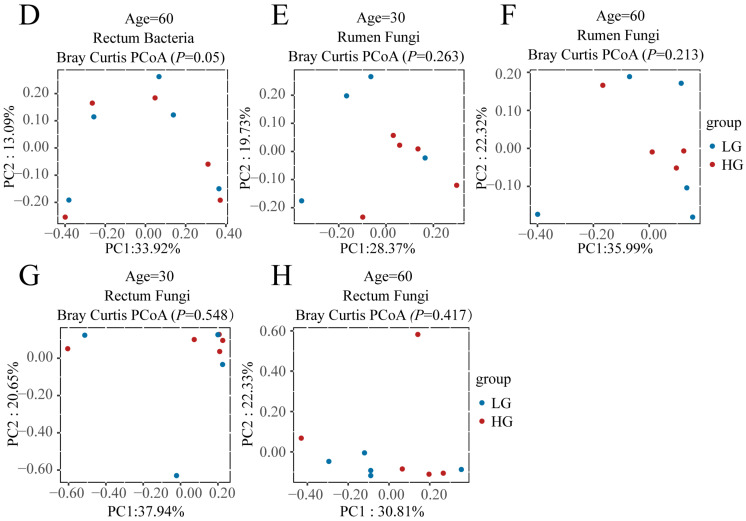
Bacterial and fungal communities and differences between the five lambs with the lower average daily gain (LG) and the five lambs with the higher average daily gain (HG) in the gastrointestinal tract before weaning. (**A**) Principal coordinate analysis (PCoA) plots of the rumen bacterial communities of LG and HG groups at 30 days of age before weaning. (**B**) PCoA plots of the rumen bacterial communities of LG and HG groups at 60 days of age before weaning. (**C**) PCoA plots of the rectum bacterial communities of LG and HG groups at 30 days of age before weaning. (**D**) PCoA plots of the rectum bacterial communities of LG and HG groups at 60 days of age before weaning. (**E**) PCoA plots of the rumen fungal communities of LG and HG groups at 30 days of age before weaning. (**F**) PCoA plots of the rumen fungal communities of LG and HG groups at 60 days of age before weaning. (**G**) PCoA plots of the rectum fungal communities of LG and HG groups at 30 days of age before weaning. (**H**) PCoA plots of the rectum fungal communities of LG and HG groups at 60 days of age before weaning.

To find what role the microbiota plays in the difference between LG and HG groups after weaning, we first compared the bacterial and fungal communities of LG and HG groups before weaning (30 and 60 days of age) in the rumen and rectum (Figure 2); the results illustrated that before weaning, there was no significant difference (*p* > 0.05) between LG and HG groups in both rumen and rectum (Figure 2A–H), except the composition of the bacterial community in the rectum tended to be different (*p* = 0.05) between the LG and HG groups (Figure 2D).

### 3.3. Differences in the Rumen and Rectum Microbiota Community of LG and HG Lambs after Weaning

**Figure 3:**
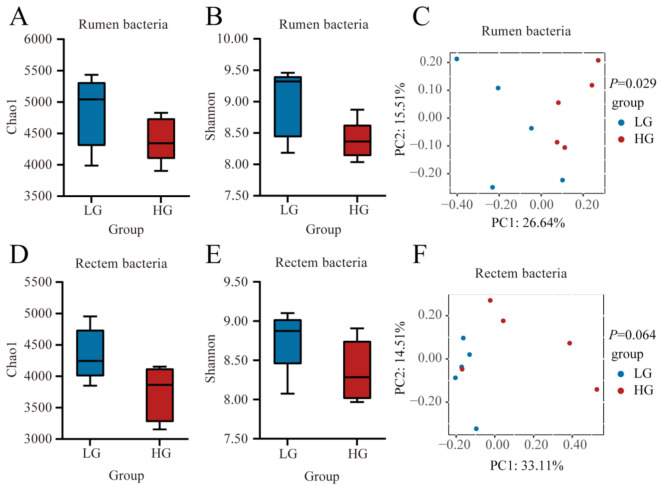
Bacterial communities and differences between the five lambs with the lower average daily gain (LG) and the five lambs with the higher average daily gain (HG) in the gastrointestinal tract at 90 days of age. (**A**) Chao 1 index and (**B**) Shannon index in the rumen. (**C**) Principal coordinate analysis (PCoA) of bacterial Bray-Curtis distance in LG and HG groups in the rumen. (**D**) Chao 1 index and (**E**) Shannon index in the rectum. (**F**) PCoA of bacterial Bray-Curtis distance in LG and HG groups in the rectum. The whiskers represent the minimum and maximum values.

After weaning, we firstly compared the Chao1 and Shannon diversity between LG and HG groups in the rumen and rectum of bacterial communities. The Chao1 index in the rumen was not affected (*p* > 0.05) by the ADG after weaning (Figure 3A). The Shannon index in the rumen tended to be higher (*p* = 0.059) in the LG group (Figure 3B). The Bray-Curtis dissimilarity analysis of the bacteria for LG and HG lambs was visualized using a PCoA plot as shown in Figure 3C. We found that the ruminal bacterial community was significantly different between LG and HG lambs as both groups show a clear separation. This was confirmed using ANOSIM analysis (*p* = 0.029). In the rectum, the Chao1 index tended to be lower (*p* = 0.053, Figure 3D) in the HG group. No significant difference (*p* > 0.05) in the Shannon index of rectum bacterial community was observed between LG and HG groups after weaning (Figure 3E). The composition of the bacterial community in the rectum tended to be different (*p* = 0.064) between LG and HG groups (Figure 3F).

**Figure 4:**
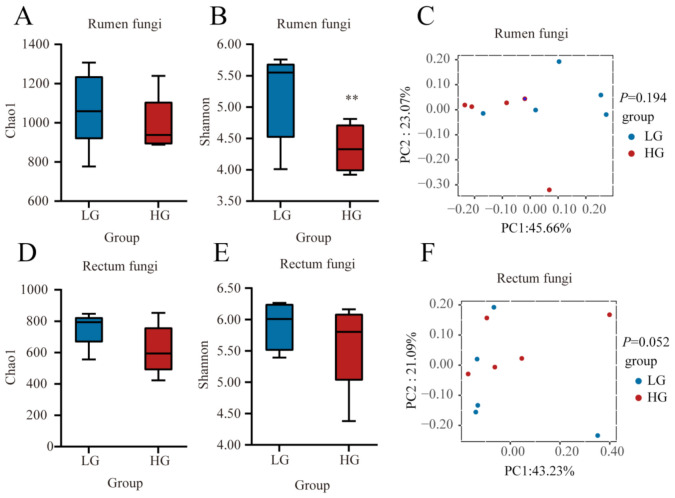
Fungal communities and differences between the five lambs with the lower average daily gain (LG) and the five lambs with the higher average daily gain (HG) in the gastrointestinal tract at 90 days of age. (**A**) Chao 1 index and (**B**) Shannon index in the rumen. (**C**) Principal coordinate analysis (PCoA) of fungal Bray-Curtis distance in LG and HG groups in the rumen. (**D**) Chao 1 index and (**E**) Shannon index in the rectum. (**F**) PCoA of fungal Bray-Curtis distance in LG and HG groups in the rectum. The whiskers represent the minimum and maximum values. ** *p* < 0.05.

Then, we compared the fungal communities in the rumen and rectum between LG and HG groups (Figure 4). No significant difference (*p* > 0.05) in the Chao1 index of rumen fungal community between LG and HG groups (Figure 4A). The Shannon index was lower (*p* < 0.05) in the HG group (Figure 4B). The composition of the fungal community in the rumen was not different (*p* > 0.05) between LG and HG groups (Figure 4C). In the rectum, no significant differences (*p* > 0.05) in the Chao 1 index and Shannon index were observed between the LG and HG groups (Figure 4D,E). The composition of the fungal community in the rectum tended to be different (*p* = 0.052) between LG and HG groups (Figure 4F).

### 3.4. The Maturity of Rumen and Rectum Microbiota in Weaned Lambs

**Figure 5:**
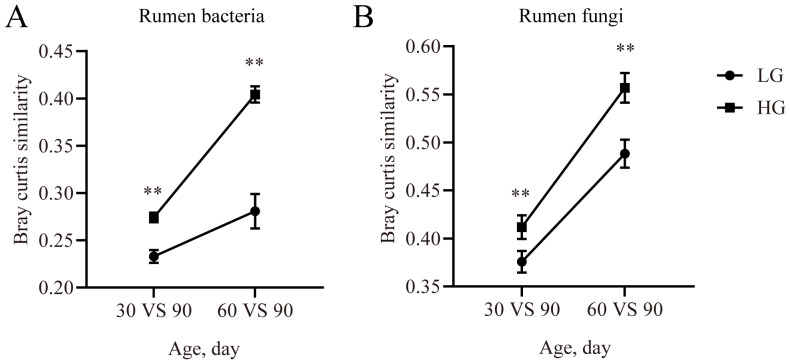

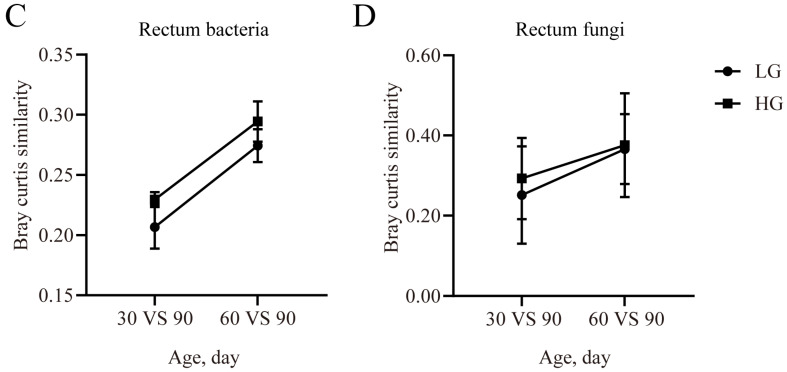
The similarity of the microbial community at different timepoints with Bray–Curtis matrix. The similarity of bacterial community between the timepoints and the 90-day-old lambs in the rumen (**A**) and rectum (**B**). The similarity of the fungal community between the timepoints and the 90-day-old lambs in the rumen (**C**) and rectum (**D**). Data are expressed as mean ± SE. ** *p* < 0.05. LG: lambs with the lower average daily gain; HG: lambs with the higher average daily gain.

In the rumen bacterial communities, the similarity was higher (*p* < 0.05) in the HG group for the 30 days vs. 90 days comparison and 60 days vs. 90 days comparison (Figure 5A). Compared with the LG group, the similarity of the rumen fungal communities was increased (*p* < 0.05) in the HG group for the 30 days vs. 90 days comparison and 60 days vs. 90 days comparison (Figure 5B).

For the rectum bacterial communities, the similarity was not affected by ADG (*p* > 0.05, Figure 5C). In the rectum fungal community, there was a tendency (0.05 < *p* < 0.1, Figure 5D) for the similarity to be higher in the HG group for the 30 days vs. 90 days comparison.

### 3.5. LEfSe Analysis for ADG-Specific Bacterial Biomarkers

**Figure 6:**
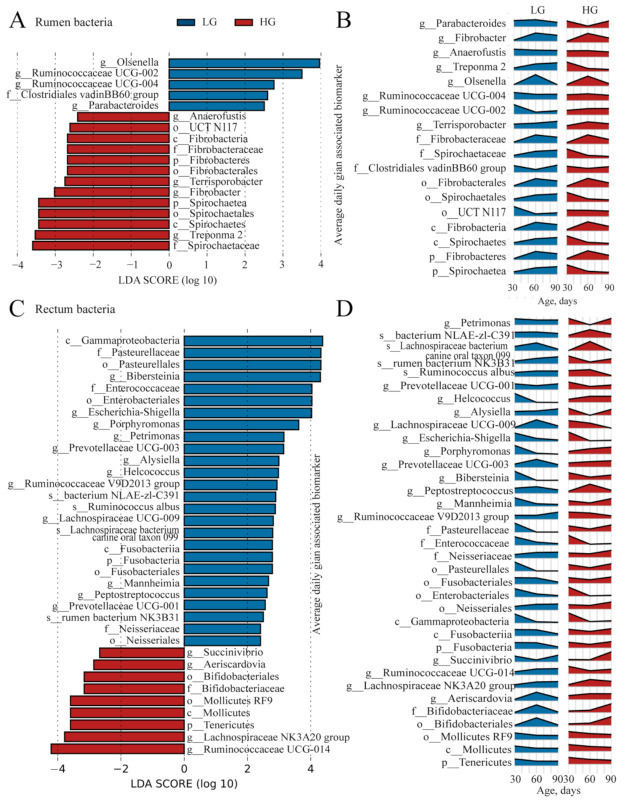
The identified bacterial biomarkers in the rumen and rectum of lambs are between the five lambs with the lower average daily gain (LG) and the five lambs with the higher average daily gain (HG). (**A**) The identified bacterial biomarkers in ruminal samples of lambs between the LG and HG groups using LEfSe analysis (LDA score > 2 and *p* < 0.05). (**B**) The changes over age show the relative abundances of the identified bacterial biomarkers in the rumen. (**C**) The identified bacterial biomarkers in rectal samples of lambs between the LG and HG groups using LefSe analysis (LDA score > 2 and *p* < 0.05). (**D**) The changes over age show the relative abundances of the identified bacterial biomarkers in the rectum.

After weaning, the difference of bacterial community between LG and HG groups increased, and the maturity of microbiota in LG and HG groups was different. Thus, to find the unique microbial biomarkers, we used the LEfSe analysis (LDA score > 2 and *p* < 0.05). The 18 unique bacterial biomarkers were identified in the rumen of weaned lambs between the LG and HG groups (Figure 6A). Four bacterial taxa, including Clostridiales vadinBB60 group, Ruminococcaceae UCG-002, Ruminococcaceae UCG-004, Parabacteroides, and Olsenella were detected as biomarkers in the rumen of LG. Two phylum Spirochaetae, Fibrobacteres and its members (Spirochaetae: Spirochaetes, Spirochaetales, Spirochaetaceae, Treponema 2; Fibrobacteres: Fibrobacteria, Fibrobacterales, Fibrobacteraceae, Fibrobacter) were detected as biomarkers in rumen of HG group. The changes in the relative abundance of the 18 biomarkers in the rumen bacterial community are presented in Figure 6B.

For rectal bacteria, we identified 35 biomarkers in the weaned lambs between the LG and HG groups (Figure 6C). The 26 biomarkers (Figure 6C) in the LG group mostly belonged to the phyla of Proteobacteria (Gammaproteobacteria, Neisseriales, Enterobacteriales, Pasteurellales, Neisseriaceae, Enterobacteriaceae, Pasteurellaceae, Mannheimia, Bibersteinia, Escherichia-Shigella, and Alysiella) and Firmicutes (Ruminococcaceae V9D2013 group, Peptostreptococcus, Lachnospiraceae UCG-009, Helcococcus, Ruminococcus_albus, and Lachnospiraceae bacterium canine oral taxon 099). Nine bacterial taxa, including Tenericutes, Mollicutes, Mollicutes_RF9, Bifidobacteriales, Bifidobacteriaceae, Aeriscardovia, Lachnospiraceae NK3A20 group, Ruminococcaceae_UCG-014, and Succinivibrio, were detected as biomarkers in the rectum of HG group. The changes in the relative abundance of the 18 biomarkers in the rumen bacterial community were presented in Figure 6D.

**Figure 7:**
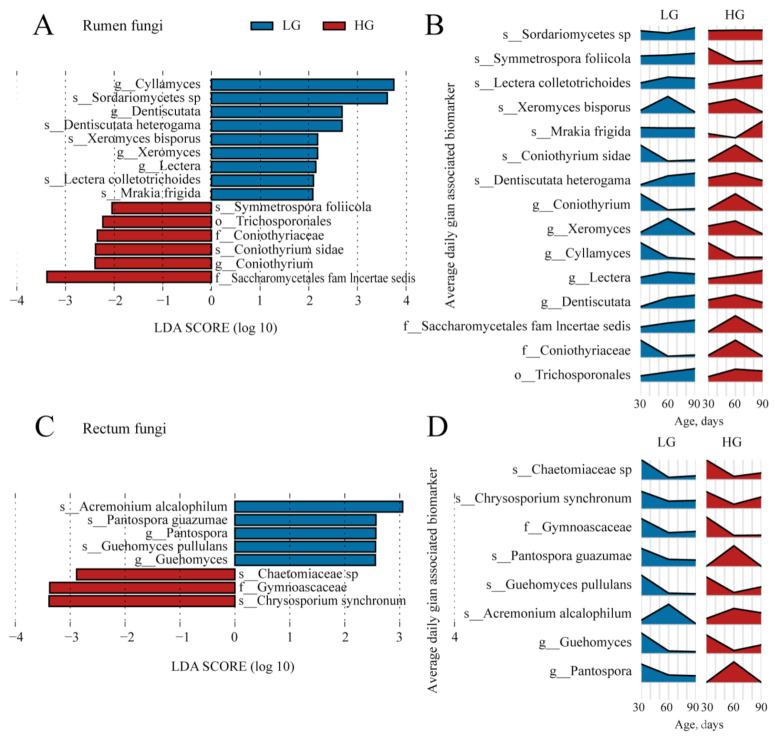
The identified fungal biomarkers in the rumen and rectum of lambs are between the five lambs with the lower average daily gain (LG) and the five lambs with the higher average daily gain (HG). (**A**) The identified fungal biomarkers in ruminal samples of lambs between the LG and HG groups using LEfSe analysis (LDA score > 2 and *p* < 0.05). (**B**) The changes over age show the relative abundances of the identified fungal biomarkers in the rumen. (**C**) The identified fungal biomarkers in rectal samples of lambs between the LG and HG groups using LefSe analysis (LDA score > 2 and *p* < 0.05). (**D**) The changes over age show the relative abundances of the identified fungal biomarkers in the rectum.

The fungal biomarkers in the ruminal microbiota between the LG and HG groups are shown in Figure 7A. Overall, 15 fungal biomarkers were identified in the rumen. Nine fungal taxa, including Dentiscutata, Lectera, Cyllamyces, Xeromyces, Dentiscutata heterogama, Mrakia frigida, Xeromyces bisporus, Lectera colletotrichoides, and Sordariomycetes sp., were identified as biomarkers in the rectum of the LG groups. Six taxa detected in the HG group belonged to phylum Basidiomycota (Trichosporonales, and Symmetrospora foliicola) and Ascomycota (Coniothyriaceae, Saccharomycetales fam Incertae sedis, Coniothyrium, and Coniothyrium sidae). The changes in the relative abundance of the 15 biomarkers in the rumen fungal community are presented in Figure 7B.

We also identified the fungal biomarkers in the rectum between the LG and HG groups (Figure 7C). Five fungal biomarkers, which specifically included Ascomycota, Basidiomycota, and their members (Ascomycota: Pantospora, Acremonium alcalophilum, and Pantospora guazumae; Basidiomycota: Guehomyces and Guehomyces pullulans), were presented in the rectum of LG group. Meanwhile, three biomarkers, which specifically belonged to the phylum Ascomycota (Gymnoascaceae, Chrysosporium synchronum, and Chaetomiaceae sp.), were detected in the rectum of the HG group. The changes in the relative abundance of the eight biomarkers in the rectum fungal community are presented in Figure 7D.

### 3.6. LefSe Analysis for ADG-Specific Bacterial Biomarkers

**Figure 8:**
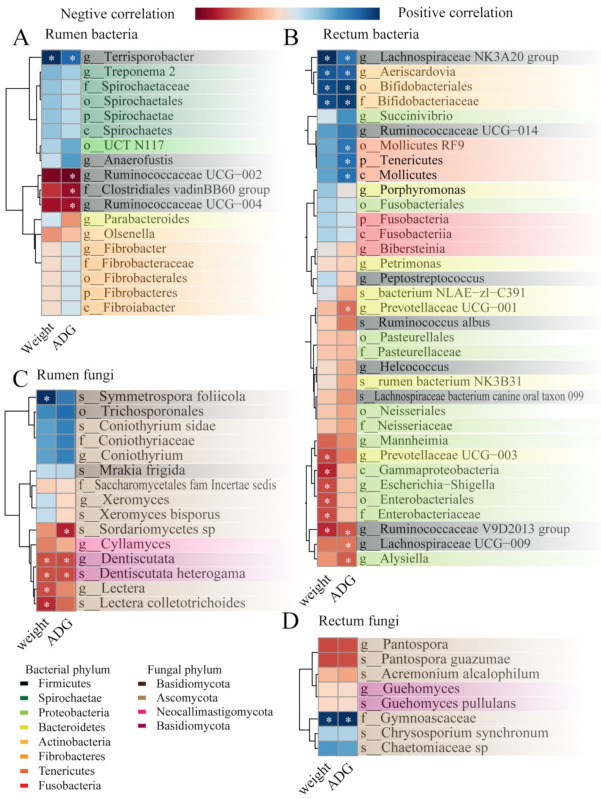
Heatmap of biomarkers (identified by LefSe analysis between the five lambs with the lower average daily gain (LG) and the five lambs with the higher average daily gain (HG) in Figure 6 and Figure 7, significantly associated with growth performance and average daily gain as determined by Spearman’s correlation analysis in the (**A**) rumen bacterial community, (**B**) rectum bacterial community, (**C**) rumen fungal community, and (**D**) rectum fungal community. * *p* < 0.05.

To investigate how the differential microbial community in the gastrointestinal tract impacts host growth, an analysis of correlations between microbial biomarkers and average daily gain after weaning was conducted, as illustrated in Figure 8.

The correlation between average daily gain and rumen bacterial community are shown in Figure 8A. The results illustrated that the final weight at 90 days old and the ADG between 60 and 90 days of age was positively (*p* < 0.05) correlated with the Terrisporobacter in the rumen. In the contrast, the ADG was negatively (*p* < 0.05) correlated with the Clostridiales adinBB60 group, Ruminococcaceae UCG-002, and Ruminococcaceae UCG-004.

We found (Figure 8B) a total of 10 taxa of the bacterial community in the rectum that were significantly (*p* < 0.05) correlated with lamb body weight at 90 days old; four taxa positively (*p* < 0.05) correlated with body weight (Lachnospiraceae NK3A20 group, Aeriscardovia, Bifidobacteriales, and Bifidobacteriaceae), and six taxa negatively (*p* < 0.05) correlated with body weight (Prevotellaceae_UCG-003, Gammaproteobacteria, Escherichia-Shigella, Enterobacteriales, Bifidobacteriaceae, and Ruminococcaceae_V9D2013_group). On the other hand, 11 taxa of the bacterial community were significantly (*p* < 0.05) correlated with ADG between 60 to 90 days of age in the rectum; of them, seven taxa were positively (*p* < 0.05) correlated with the ADG (Lachnospiraceae_NK3A20_group, Aeriscardovia, Bifidobacteriales, Bifidobacteriaceae, Mollicutes_RF9, Tenericutes, and Mollicutes) and four taxa negatively (*p* < 0.05) correlated with the ADG (Prevotellaceae_UCG-001, Ruminococcaceae_V9D2013_group, Lachnospiraceae_UCG-009, and Alysiella).

Among the 15 fungal biomarkers identified in the rumen above, five biomarkers showed significant Spearman correlations (Figure 8C, *p* < 0.05) with the body weight at 90 days old (positive correlation: Symmetrospora_foliicola; negative correlation: Dentiscutata, Lectera, Dentiscutata_heterogama, Lectera_colletotrichoides); meanwhile, three biomarkers showed negatively correlations (*p* < 0.05) with the ADG between 60 to 90 days of age (Dentiscutata, Dentiscutata_heterogama, Sordariomycetes_sp).

Furthermore, the correlation between average daily gain and rectum fungal community are shown in Figure 8D. The Gymnoascaceae was significantly related (*p* < 0.05 to the body weight at 90 days old and the ADG between 60 and 90 days of age).

## 4. Discussion

The present study aimed to determine the diverse microbial communities in the rumen and rectum of lambs and to examine the association of average daily gain with the microbiota populations in the gastrointestinal tract. We found that ADG was correlated with the rumen and rectum microbial community. Some taxa were significantly correlated with the ADG of weaned lambs, indicating that these taxa may play an important role in altering ADG. Our hypothesis that the rumen and rectum microbial community of low ADG lambs is different from high ADG lambs has been supported by the results of this study, which may provide a framework for improving the growth performance of weaned lambs.

Understanding the compositional and functional differences in the gut microbiota can lay the foundation to connect these differences to animals’ health [33]. Our results demonstrated that before weaning, the body weight of 30 and 60 days old, the ADG between 30 and 60 days of age, and the rumen and rectum microbial communities at 30 and 60 days of age did not differ between LG and HG groups. However, one month after weaning, the body weight and the ADG dramatically increased in the HG group, and the microbial communities were different between the LG and HG groups. During weaning, animals are shifted to a solid feed and separated from their mother, which causes the gut microbiota to reassemble [34]. The sudden changes may lead to immune responses, affect nutrient absorption, and injure barrier function [35,36]. Our study found that though the microbial communities were not different before weaning, the maturity of both bacterial and fungal communities in the rumen was increased in the HG group compared to the LG group. The difference in the maturity of the LG and HG groups demonstrates that the microbiota in the HG groups was more similar to that of mature lambs, which may accelerate the changes of microbiota to adapt to digest solid food, thus, helping to reduce the weaning stress of lambs, improving the growth performance.

In addition, the analysis of the alpha diversity showed that the microbial communities of the LG group had a more diverse microbial community at 90 days of age. The gut microbial diversity plays a vital role in keeping a steady microbial community [37]; high microbial diversity is closely linked to strong stability and resistance in response to the environment [38]. A study in beef cattle suggest that a more ‘diverse rumen’ was capable of fermenting a wider range of substrates but had a lower feed conversion ratio; in contrast, a ‘simple rumen’ possibly generates more specific products that can be more efficiently absorbed and utilized by the host [39]. The study in residual feed intake had similar results that the efficient cows had less diverse microbiome to produce molecules serving as energy [40]. The composition of the microbiome affects the ratio of end products during fermentation: an ‘inefficient rumen’ produces unusable end products such as methane [41]. Therefore, we speculate that facing the disturbance caused by weaning, the microbial community of the LG group with more ‘diverse rumen’ require more efforts, leading to lower maturity, different microbial communities, and lower feed efficiency, finally causing lower ADG. Diversity is important in all ecosystems to promote stability and performance [42]. Microbiota diversity may become a new biomarker or indicator of health [26]. While the roles of different types of microbiota diversity in host health remain to be defined, it is possible that the microbial diversity may be related to the growth performance of lambs.

The first step in understanding the relationship between gut microbes and their host is to characterize the differences associated with performance [33]. The microbiome plays a critical role in providing nutrition to the host animal, thus influencing ruminant performance [43]. For pre-weaned goats, the Spirochaetae gradually increased with age [44], and it was more abundant in older animals in the rumen [45], suggesting that Spirochaetae play an essential role in ruminants maturing process. The phylum Spirochaetae, including its members (Spirochaetes, Spirochaetales, Spirochaetales, Spirochaetaceae, and Treponema 2), had a higher relative abundance in HG groups in the rumen. In the present study, the phylum Fibrobacteres and its members, including Fibrobacteria, UCT N117, Fibrobacterales, Fibrobacteraceae, and Fibrobacter were distinct signature taxa in the rumen of the HG groups. Several studies showed that Fibrobacteres were found predominantly in the rumen and could degrade plant-based cellulose [46]; this was associated with the fiber content in different diets, especially in hay-fed animals [47]. Meanwhile, the Treponema spp. which was considered to be an active decomposer of plant polysaccharides in herbivores [48,49], was one of the distinct signature taxa in the rumen of HG groups. Our results indicated that the rumen bacterial community in the HG group was more similar to the mature lambs and contains more fiber-digested bacteria. Bifidobacteriales and their members are one of the major components in the microbiota that are suggested to function in maintaining health for humans [50], pigs [4], and steers [51]. Bifidobacteriales can form a biofilm which plays a vital role in preventing pathogen invasion and stimulating the host immune functions [52]; it was an important biomarkers for healthy gut micro-ecosystems [53]. A previous study investigated the higher prevalence of butyrate-producing bacteria belonging to families Lachnospiraceae (Lachnospiraceae NK3A20 group) in healthy ruminants [26], which may enhance the host’s energy harvesting capacity [54]. In the present study, Bifidobacteriales and their members, and genera Lachnospiraceae NK3A20 group were positively correlated with ADG and live weight after weaning in the rectum. Meanwhile, Fusobacteria was abundant in the childhood of humans [55,56], was enriched in the steers which had relatively lower residual feed intake [57], and also was a distinct signature taxon in the rectum of the LG group. Family Enterobacteriaceae tends to have more potential pathogenic bacteria such as the genus Escherichia-Shigella [58], which is a primary initiator of diarrhea in calves. Weaning stress may be associated with gastrointestinal disorders with post-weaning diarrhea major caused by Escherichia-Shigella [59]. The Escherichia-Shigella was a distinct signature taxon in the rectum of the LG group, indicating that weaned lambs may be more susceptible to intestinal infections, which may lead to diarrhea. Thus, we speculated that compared to the LG group, the rumen and rectum bacterial communities in the HG groups may help the lambs to degrade solid food and simultaneously improve the health of the lambs by significantly reducing potential pathogenic bacteria diarrhea. Our results suggest the possibility to improve host growth performance using the specific microbiota, however, the function of these bacteria needs further study.

In addition, we revealed differences in the rumen and rectum fungal composition in lambs with different ADG using LEfSe, which provided a foundation for the modification of the gastrointestinal microbiota in ruminants in early life. During the distinct signature taxa in the rumen, the genus Xeromyces and species Mrakia frigida all belonged to the LG group. The genus Xeromyces was observed to be negatively correlated with the concentration of acetate in weaned piglets [60]. For the rectum, a previous study found the genus Acremonium was one of the age-related genera, indicating that the species of fungi are important contributors to gastrointestinal function in sheep [61]. The genus Acremonium was the signature taxa in the rectum of the LG group, suggesting the hindgut in the LG group was developing. Though we found several specific taxa, and some of them were significantly correlated with body weight and ADG, the function of these fungal taxa remains unclear. Our results also suggest that further research should focus on the function of the neglected microbes in the previous studies in the gastrointestinal tract.

## 5. Conclusions

Our results demonstrated that though the rumen and rectum microbiota did not differ between the LG and HG groups before weaning, some specific taxa were already persisting and affected the maturity of lambs. The signature taxon in the HG group was more similar to the mature lambs, contains more fiber-digested bacteria and health indicators. On the other hand, the signature taxon in the LG groups contains diarrhea indicators. We speculated that compared to the LG group, the microbial communities in the HG groups may help the lambs to degrade solid food and simultaneously improve the health of the lambs by significantly reducing potential pathogenic bacteria diarrhea, suggesting the possibility to improve host growth performance using the specific microbiota. The function of the specific bacteria needs further study. Although our study was only a preliminary investigation, the results would be helpful in developing our understanding of the gastrointestinal microbiota and provides a basis for future work on the function of gastrointestinal microbiota in ruminants.

## 6. Limitations

The limitation of this study is that the nutrient intake of lambs was unknown. Especially in the pre-weaning period, the nutritional level of suckling lambs was unknown, because we did not use an artificial rearing system before weaning, which will cause a different maturing process in gastrointestinal microbiota due to the various nutrient intake in the milk, as well as their mothers’ microbiome. Though the diet was the same between different groups, the feed intake before or after weaning will all influence the average daily gain and the maturation in the gastrointestinal of lambs. In the present study, we found several distinct microbial taxa related to the ADG of lambs, but we did not investigate the factors that will influence the microbial community.

In addition, strong conclusions cannot be made where the *n* = 5 for the microbial studied group. In our study, we randomly selected five lambs to detect the gastrointestinal microbiota. Though the number of lambs meets the requirement of statistical analysis, the small number of replicates may lead to statistical error. Further works will be required to investigate.

## Data Availability

The datasets analyzed for this study can be found in the Genome Sequence Archive repository (http://gsa.big.ac.cn) accessed on 19 June 2020. Please see the accession numbers PRJCA002881 for more details.

## Supplementary Materials

The following supporting information can be downloaded at: https://www.mdpi.com/article/10.3390/microorganisms11020348/s1, Table S1: Nutrient composition of experimental diets of Hu sheep (dry matter basis).
