# Supplementary material for: Average Daily Gain in Lambs Weaned at 60 Days of Age Is Correlated with Rumen and Rectum Microbiota
Source: Microorganisms. 2023 Jan 30;11(2):348. doi: 10.3390/microorganisms11020348 (PMC9966089; doi:10.3390/microorganisms11020348)
Supplement: Supplementary file 1 [file microorganisms-11-00348-s001.zip › microorganisms-2155702-supplementary.pdf]

Average daily gain in lambs weaned at 60 days of age is correlated with rumen and rectum microbiota

Xuejiao Yin[1†], Chunhui Duan[1†], Shoukun Ji[1], Peizhi Tian[1], Sisi Ju[1], Hui Yan[1], Yingjie Zhang[1*], Yueqin Liu[1]

[1]College of Animal Science and Technology, Hebei Agricultural University, Baoding 071000, P. R. China

† These authors have contributed equally to this work.

*** Correspondence:**

Yingjie Zhang

zhangyingjie66@126.com

Running title: Correlation of gut microbiota to average daily gain.

Supplementary Table S1. Nutrient composition of experimental diets of Hu sheep (dry matter basis)

Items[1], %	Starter	TMR
Ingredients		
Cornstalk		40.00
Corn	53.20	35.50
Soybean meal	9.10	15.00
Wheat bran	2.00	7.20
Soybean skin	6.20	
Beet pulp	4.70	
Extruded Soybean	8.50	
Fermented soybean meal	9.60	
Soybean oil	2.20	
Whey powder	2.00	
Limestone	1.20	0.30
$CaHPO_4$		0.20
NaCl	0.30	0.80
Premix[2]	1.00	1.00
Total	100.00	100.00
Chemical compositions		
Dry matter	95.83	77.20
Gross energy, MJ/kg DM	17.56	15.07

Crude protein	21.20	14.61
Neutral detergent fiber	16.44	37.60
Acid detergent fiber	7.04	21.81
Calcium	0.74	0.70
Phosphorus	0.48	0.43

[1]Chemical composition measured by AOAC method. TMR: Total Mixed Rations.

[2]Premix containing: 17456 IU/kg of vitamin A, 50 mg/kg of vitamin E, 3740 IU/kg of vitamin D, 98.70 mg/kg of Fe, 72.90 mg/kg of Zn, 57.4 0 mg/kg of Mn, 15.94 mg/kg of Cu, 0.33 mg/kg of Se, 1.30 mg/kg of I and 0.39 mg/kg of Co.
